# Photocatalytic H_2_ Production Using Pt-TiO_2_ in the Presence of Oxalic Acid: Influence of the Noble Metal Size and the Carrier Gas Flow Rate

**DOI:** 10.3390/ma7107022

**Published:** 2014-10-17

**Authors:** Ákos Kmetykó, Károly Mogyorósi, Viktória Gerse, Zoltán Kónya, Péter Pusztai, András Dombi, Klára Hernádi

**Affiliations:** 1 Research Group of Environmental Chemistry, Institute of Chemistry, Faculty of Sciences and Informatics, University of Szeged, H-6720 Szeged, Tisza L. krt. 103., Hungary; E-Mails: kmetykoakos@chem.u-szeged.hu (A.K.); k.mogyorosi@chem.u-szeged.hu (K.M.); gerse.viktoria@mailbox.hu (V.G.); hernadi@chem.u-szeged.hu (K.H.); 2 Department of Applied and Environmental Chemistry, Faculty of Sciences and Informatics, University of Szeged, H-6720 Szeged, Rerrich tér 1., Hungary; E-Mails: konya@chem.u-szeged.hu (Z.K.); peter.pusztay@gmail.com (P.P.)

**Keywords:** TiO_2_, H_2_ production, Pt nanoparticles, size dependent activity, oxalic acid

## Abstract

The primary objective of the experiments was to investigate the differences in the photocatalytic performance when commercially available Aeroxide P25 TiO_2_ photocatalyst was deposited with differently sized Pt nanoparticles with identical platinum content (1 wt%). The noble metal deposition onto the TiO_2_ surface was achieved by *in situ* chemical reduction (CRIS) or by mixing chemically reduced Pt nanoparticle containing sols to the aqueous suspensions of the photocatalysts (sol-impregnated samples, CRSIM). Fine and low-scale control of the size of resulting Pt nanoparticles was obtained through variation of the trisodium citrate concentration during the syntheses. The reducing reagent was NaBH_4_. Photocatalytic activity of the samples and the reaction mechanism were examined during UV irradiation (λ_max_ = 365 nm) in the presence of oxalic acid (50 mM) as a sacrificial hole scavenger component. The H_2_ evolution rates proved to be strongly dependent on the Pt particle size, as well as the irradiation time. A significant change of H_2_ formation rate during the oxalic acid transformation was observed which is unusual. It is probably regulated both by the decomposition rate of accumulated oxalic acid and the H^+^/H_2_ redox potential on the surface of the catalyst. The later potential is influenced by the concentration of the dissolved H_2_ gas in the reaction mixture.

## 1. Introduction

Hydrogen is one of the cleanest energy sources on Earth. The field of heterogeneous photocatalysis can help convert the energy of sunlight to chemical energy via the production of H_2_ gas from water [1,2]. TiO_2_ photocatalysts can be excited within the UV spectral range, although in most cases bare titanias have low efficiency for photocatalytic water splitting. The overvoltage of H_2_ evolution and the recombination rate of electron-hole pairs can be lowered by depositing noble metal particles onto the surface of TiO_2_ [3]. In the literature Pd [4,5], Pt [6,7,8], Ru [9], Rh [4], Au [10,11] and Ag [12] are used to enhance the photocatalytic efficiency of H_2_ generation on TiO_2_. Noble metal particles can be deposited onto the catalyst’s surface by either sonochemical method [13], photoreduction [14,15,16], or chemical reduction (in liquid phase with e.g., hydrazine [17,18] or sodium borohydride [19,20]).

The size and amount of noble metal particles on the TiO_2_ surface are very important factors that can affect the overall activity of the catalyst. Larger quantities of metal particles on the titania can block the active sites and large number of metal deposits can decrease charge carrier space distance which leads to increased electron-hole recombination rates [5,21]. In most cases, noble metal content of 1 wt% [22,23,24] or below [25,26] is considered to be optimal. The size and dispersion of noble metal nanoparticles can determine the number of these particles per one TiO_2_ particle. Theoretically, larger Pt particles form fewer noble metal islands on TiO_2_ than smaller ones do if noble metal content is identical. The electrostatic effect of noble metal islands is limited to a group of nearby TiO_2_ particles. According to this phenomenon, smaller sized noble metal particles are desirable theoretically. However, if the deposit size is too small, it is hard to establish sufficient electrical contact for efficient charge transfer [27]. The size of noble metal nanoparticles can be gently controlled by adding surfactants to the metal precursor containing solution before the reduction process. The dimensions of the so forming nanoparticles are dependent upon the surfactant’s quality and concentration [28,29]. In regard to the important influence of metal nanoparticle size on the catalyst’s activity, developing an easily adjustable synthesis method with good reproducibility and using additives that can be readily removed after reduction are substantial steps to make the process economic. Noble metal nanoparticles below 10 nm deserve special attention in these experiments.

During the photocatalytic reactions, the presence of easily oxidizable organic compounds in the reaction mixture (instead of using only pure water) is desirable. These electron donors (so called sacrificial reagents) react irreversibly with the photoinduced holes resulting in suppressed electron-hole recombination rates. In numerous papers, different kinds of alcohols (especially methanol) are used as hole scavengers [30,31,32,33]. Some carboxylic acids also can act like effective hole scavengers, such as formic acid [34], chloroacetic acid [35] or oxalic acid [16]. The enhancement in H_2_ production also depends on the concentration of the sacrificial reagent. While there are numerous publications about enhancing the hydrogen evolution efficiency by depositing Au on TiO_2_, Pt loaded TiO_2_ might be a more efficient photocatalyst for H_2_ production due to its better ability to act as an electron trap as proved recently [36].

## 2. Experimental Section

1 wt% (*m*_Pt_/*m*_TiO2_) Pt-modified TiO_2_ photocatalysts were prepared containing differently sized Pt nanoparticles. Particle size was gently adjustable by varying only one factor during the syntheses: the concentration of the surfactant. Thus, photocatalytic activities of these catalysts are well comparable according to the Pt particle size. Effect of the noble metal particle size on the photocatalytic H_2_ production was thoroughly investigated in the range of 2.5–4.5 nm.

### 2.1. Catalyst Preparation

Aeroxide P25 (Evonik Industries AG, Essen, Germany) TiO_2_ (*D*_av_ = 25.4 nm, 90% anatase and 10% rutile) was used as bare catalyst. All the syntheses and photocatalytic tests were carried out in Millipore MilliQ ultrapure water as medium. Pt nanoparticles were deposited onto the TiO_2_ surface by chemical reduction (CR) or photoreduction (PR) methods.

#### 2.1.1. Chemical Reduction Method

During this synthesis method, different concentrations of trisodium citrate (2.50 × 10^−4^ M; 1.88 × 10^−4^ M; 1.25 × 10^−4^ M and 0.63 × 10^−4^ M) were used. Citrate anions, which help stabilize the Pt nanoparticles in the growing step after nucleation, provide good conditions for size focusing and synthesizing monodisperse noble metal nanoparticles. The reaction mixture was thermostated at 20 °C. Trisodium citrate was added to the TiO_2_ suspension (*c*_TiO2_ = 5 g/L), followed by H_2_PtCl_6_ (final concentration: 2.5 × 10^−4^ M). Finally, freshly prepared, ice-cold NaBH_4_ solution was added as a reducing agent (final concentration: 3 × 10^−3^ M). The suspension immediately turned grey. As the reduction took place in the presence of TiO_2_, this procedure was designated CRIS (chemical reduction, *in situ*). After 1 h the suspension was washed by centrifugation, re-suspending the particles in oxalic acid solution (5.0 × 10^−2^ M) to improve the sedimentation and to get rid of the chloride and sodium remnants. The final suspension was used freshly for photocatalytic test without any further processing.

Other portion of Pt-modified TiO_2_ catalysts were prepared by mixing the previously chemically reduced Pt sol with the TiO_2_ suspension (chemically reduced, sol-impregnated samples, CRSIM). The washing procedure was the same as for the *in situ* prepared Pt-TiO_2_.

#### 2.1.2. Photoreduction Procedure

To promote the photoreduction of Pt(IV) ions, oxalic acid (OA) was added to the reaction mixture as hole-scavenger organic compound (PROA samples). UV photons excite the TiO_2_, the organic compound is oxidized by the holes, and at the same time Pt(IV) is reduced by the excited electrons.

TiO_2_ was suspended in water (5 g/L), and H_2_PtCl_6_ was added to achieve a concentration of 2.5 × 10^−4^ M (1 wt% Pt on TiO_2_), followed by the hole-scavenging organic compound (*c*_OA_ = 5.0 × 10^−2^ M). The suspension was next subjected to UV irradiation for 2 h to allow photoreduction of the noble metal ions. Within 1–5 min, there was a characteristic color change, from white to gray, which indicated the formation of Pt nanoparticles. Then, the suspension was washed by centrifugation as described in Section 2.1.1. For the photocatalytic experiment, the redispersed catalyst was used immediately. During this synthesis method, the reduction process is much slower than in the case of chemical reduction; therefore, slightly bigger Pt nanoparticles are formed.

### 2.2. Characterization of the Catalysts

#### 2.2.1. Spectrophotometry

The UV-VIS spectra of Pt sols were measured in 1 cm quartz cells in an Agilent 8453 diode array spectrophotometer (Agilent Technologies, Santa Clara, CA, USA), with Millipore MilliQ ultrapure water as blank reference.

#### 2.2.2. Transmission Electron Microscopy (TEM)

The average size of the Pt nanoparticles deposited on Aeroxide P25 TiO_2_ was calculated according to TEM images recorded with a 200 kV Fei Tecnai G2 20 Xtwin instrument (FEI, Hillsboro, OR, USA). The catalyst samples were investigated immediately after preparation.

#### 2.2.3. ICP Measurements

Concentration of Pt that may have been detached or dissolved from the surface was determined by ICP-MS spectrometry (Model 7700x, Agilent Technologies, Santa Clara, CA, USA), equipped with a Micromist nebulizer, Peltier-cooled spray chamber and an integrated autosampler (G3160B, Agilent). Prior their use, all labware (flasks, autosampler vials, *etc.*) were thoroughly cleaned using trace quality HNO_3_ (Suprapur, Merck KGaA, Darmstadt, Germany) and HCl. External calibration was used based on the signal from the ^195^Pt isotope, with a good linear fit (*r*^2^ = 0.9998). Standard solutions were diluted from Pt stock solution (ICP-MS standard, Certipur, Merck) using Millipore MilliQ quality deionized water.

### 2.3. H_2_ Production Measurements

The suspension of the freshly-prepared, washed catalyst in 50 mM oxalic acid solution was poured into a glass reactor (total volume: 150 mL), surrounded by ten 15 W UV lamps (λ_max_ = 365 nm). The well-stirred suspension (*c*_catalyst_ = 1 g/L) was purged with N_2_ at a flow rate of 50 mL/min (in the majority of experiments) to ensure O_2_-free conditions. The reactor was connected through a PTFE tube to a Hewlett Packard 5890 gas chromatograph fitted with 5Å molecular sieve column and a thermal conductivity detector. Gas samples were taken with a 2 mL sampling valve, every 10 min in the first hour of the experiment and every 20 min in the second hour. The rate of H_2_ evolution was calculated with regard to the GC calibration (carried out with certified 5% H_2_:N_2_ gas) and the N_2_ flow rate.

The reactor was characterized by actinometric measurement according to the iron oxalate method (*I*_A_ = 6.35 × 10^−5^ Einstein/sL).

### 2.4. UV Decomposition of Oxalic Acid

These experiments were carried out under the same conditions as H_2_ production measurements, but liquid samples were taken from the suspensions at predetermined intervals during the reaction, and the residual oxalic acid concentration was measured. After centrifugation and filtration with a Whatman Anotop 25 0.02 μm syringe filter, the HPLC measurements were performed on a Merck Hitachi device fitted with an L-4250 UV-VIS detector and a GROM Resin ZH 8 μm column.

### 2.5. Adsorption of Oxalic Acid on the Catalyst

Oxalic acid adsorption was investigated on bare and platinized TiO_2_. The applied oxalic acid concentrations were in the range of 0.1–50 mM. The 1 g/L suspensions were thermostated at 25 °C and stirred for 4 h in the dark. Then the samples were filtered using a Whatman Anotop 25 0.02 μm syringe filter and the residual oxalic acid concentration was determined by HPLC detailed in Section 2.4.

## 3. Results and Discussion

### 3.1. Reduction of Pt(IV)

**Figure 1:**
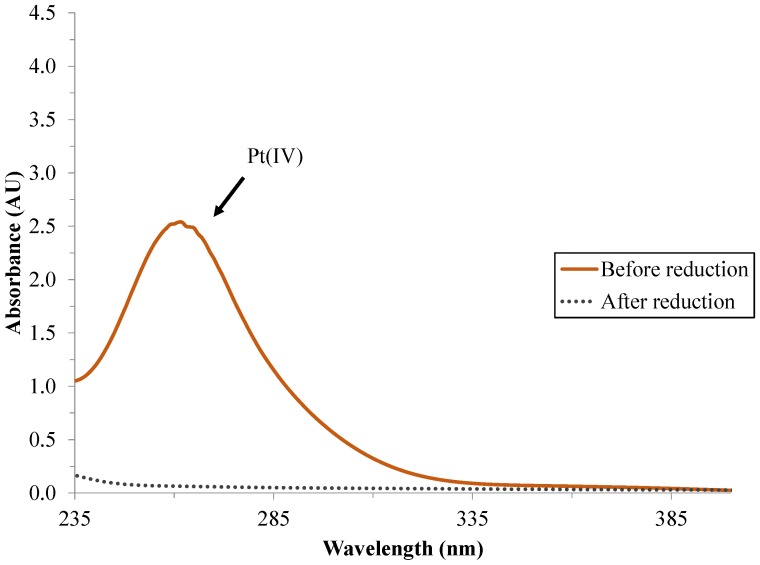
Absorption spectra of Pt(IV) solution and Pt sol reduced by NaBH_4_.

Pt(IV) ions have a light absorption peak at ~258 nm [37]. This peak completely disappeared after the chemical reduction procedure, confirming that Pt(IV) ions were reduced to Pt(0) during the 1 h reaction time (Figure 1).

It should be mentioned that with all procedures, the supernatants of Pt-TiO_2_ suspensions were colorless, which indicates that the Pt nanoparticles were all well stabilized on the surface of TiO_2_.

### 3.2. Size of the Pt Particles on TiO_2_

**Figure 2:**
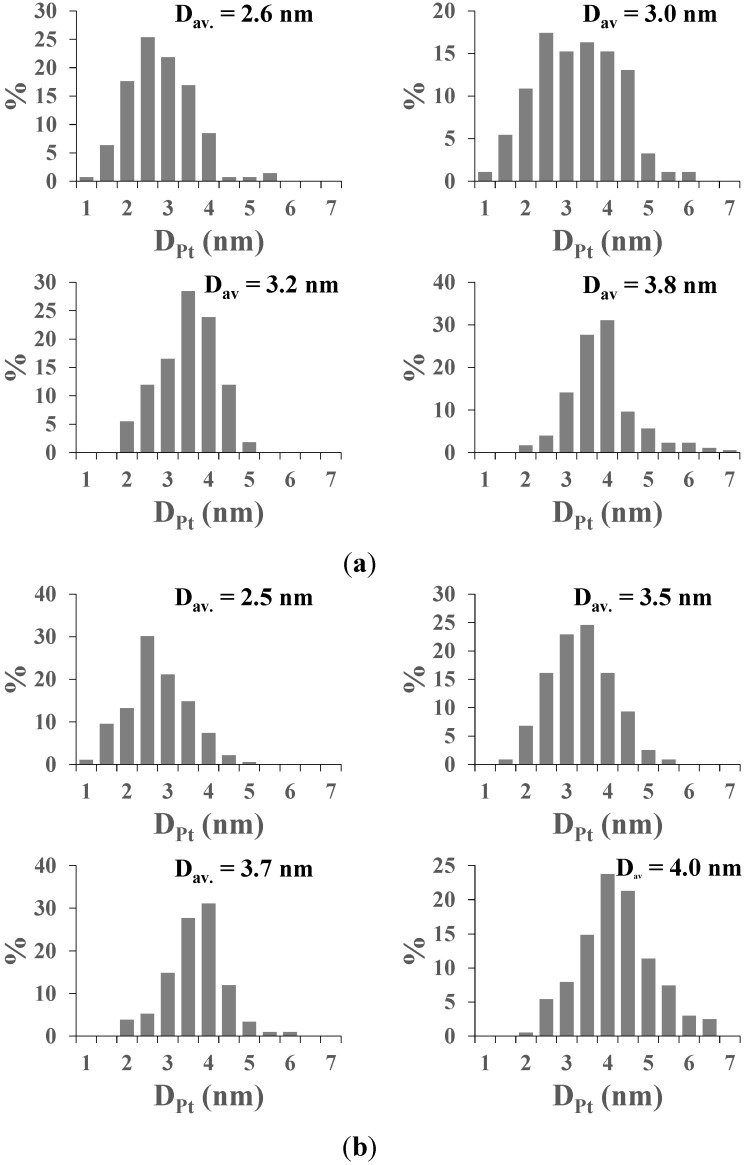
Relative frequency of the formed Pt nanoparticles according to the particle size. The concentration of the stabilizing agent during the syntheses was varied in four equal steps between 2.5 × 10^−4^ M and 0.63 × 10^−4^ M. (**a**) *in situ* chemically reduced (CRIS) samples and (**b**) chemically reduced, sol impregnated (CRSIM) samples.

By measuring ~200 particles of Pt on each sample’s TEM images, the size of the noble metal nanoparticles appeared to be dependent on the stabilizing trisodium citrate concentration during the chemical reduction process. Larger average Pt nanoparticle size was obtained with lower stabilizer concentration. The size distribution of the formed Pt nanoparticles is shown in Figure 2. The Pt particles were mainly spherical.

### 3.3. Effect of Surface Pt Nanoparticles on the Oxalic Acid Adsorption

**Figure 3:**
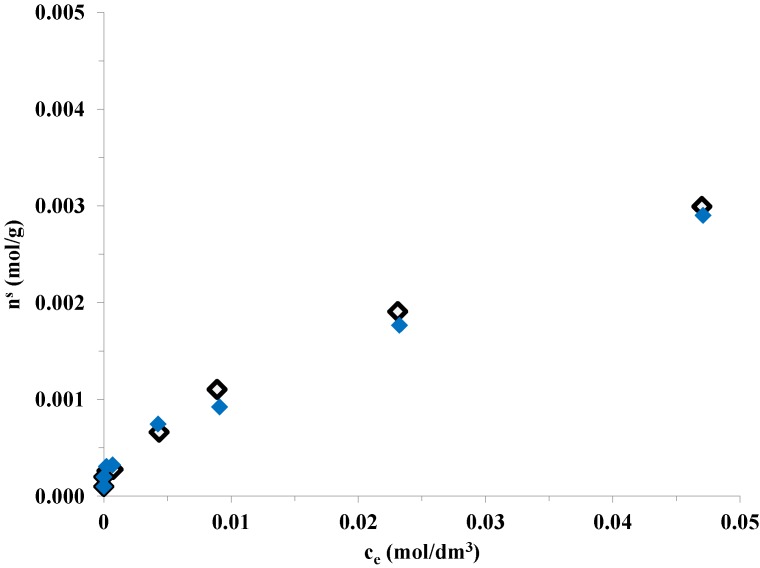
Adsorption isotherms of oxalic acid on bare Aeroxide P25 (◊) and 1 wt% Pt-modified Aeroxide P25 (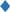) TiO_2_ photocatalysts at 25.0 °C.

Oxalic acid is adsorbed on the TiO_2_ surface by dissociative adsorption [38]. The adsorption properties of oxalic acid were investigated on bare and Pt modified catalysts in 1 g/L suspensions at 25 °C. The bare Aeroxide P25 TiO_2_ adsorbed almost the same amount of oxalic acid on its surface than did the 1 wt% Pt-modified Aeroxide P25 (Figure 3).

At the initial oxalic acid concentration applied in the H_2_ production experiments (50 mM), all of the binding sites on the TiO_2_ surface were likely to be covered by oxalate ions (adsorbed amount is 3 mmol/g). It can be concluded that modifying the titania surface with 1 wt% Pt nanoparticles does not affect considerably the amount of binding sites on the TiO_2_ surface within experimental error.

### 3.4. Photocatalytic Experiments

#### 3.4.1. H_2_ Production from Oxalic Acid Solution

H_2_ evolution was measured in the presence of 50 mM oxalic acid in N_2_-purged suspensions. Relatively high oxalic acid concentration was applied in order to keep the substrate concentration decrease negligible: during the measurement oxalic acid can decompose mostly to CO_2_ and H_2_ under O_2_-free conditions [16]. Moreover, the initial concentration of oxalic acid was at least 200 times higher than the citrate concentration used during the syntheses. However, most of the citrate ions were presumably washed out during centrifugation. There was a significant decrease in H_2_ production in the first 40 min of irradiation using any Pt-TiO_2_. After this period, the H_2_ production curves reached saturation level and the H_2_ evolution rate subsequently remained almost constant for the rest of the experiment with all Pt-modified TiO_2_ catalysts.

**Figure 4:**
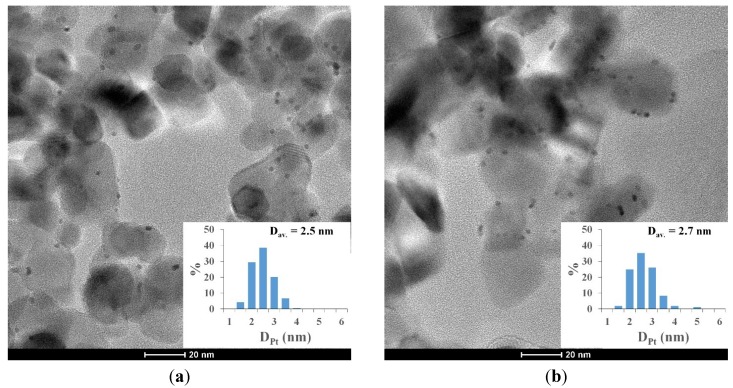
TEM images of Pt-TiO_2_ (Pt was stabilized with 2.5 × 10^−4^ M citrate during synthesis) (**a**) before (*D*_Pt, av_ = 2.5 nm) and (**b**) after 2 h of UV irradiation (*D*_Pt, av_ = 2.7 nm).

Some experiments were performed to find an explanation for this phenomenon. We investigated the possibility of the change in Pt nanoparticle size during the photocatalytic reaction. Comparing the average platinum particle size in TEM images before the experiment and after 2 h of UV-irradiation in oxalic acid solution, we determined that irradiating these catalysts does not affect considerably the average particle diameter and the size distribution of noble metal nanoparticles (Figure 4).

Monitoring the platinum concentration in the solution phase by ICP measurement, we did not observe any significant Pt dissolution from the surface of the catalyst during the experiment (Table 1).

**Table 1:** Concentration of platinum in the supernatant (dissolved from TiO_2_ surface) as a function of irradiation time (total concentration of platinum in the suspension is 10,000 ppb).

Irradiation time (min)	*c*_Pt_ (ppb)
0	1.87
10	0.60
20	0.67
40	0.42
60	0.25
80	0.38
120	0.34

**Figure 5:**
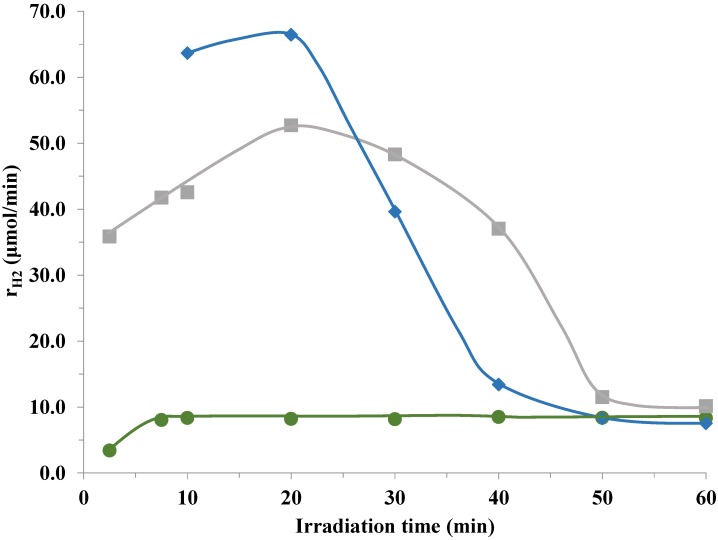
Differences in initial H_2_ evolution rates according to the flow rate of the purging N_2_ gas (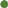 25 cm^3^/min, 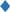 50 cm^3^/min, 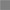 138 cm^3^/min).

The platinum concentration in the solution phase was 1.87 ppb and remained below 1 ppb during irradiation which is less than 0.01% of the total platinum concentration in the suspension. Therefore, the disappearance of metallic platinum from the surface of catalyst is insignificant. However, changing the flow rate of purging gas had an important influence over the hydrogen formation at the first stage of experiments. The initial maximum of H_2_ evolution prolonged by increasing and disappeared by decreasing the flow rate of N_2_, as is shown in Figure 5.

It is theoretically plausible that at the first stage of hydrogen formation the reaction is regulated electrochemically by the redox potential of H-electrode formed by the hydrogen absorption-desorption rate in platinum [39]. With a higher N_2_ flow, the hydrogen absorption is limited, but at low flow rates, a steady state redox potential can set much faster. Thus, no initial peak in the hydrogen evolution curve was observed at 25 cm^3^/min N_2_ flow, while there is a prolate maximum curve when adjusting the carrier gas to higher flow rates.

#### 3.4.2. Effect of Pt Particle Size on Photocatalytic H_2_ Generation

**Figure 6:**
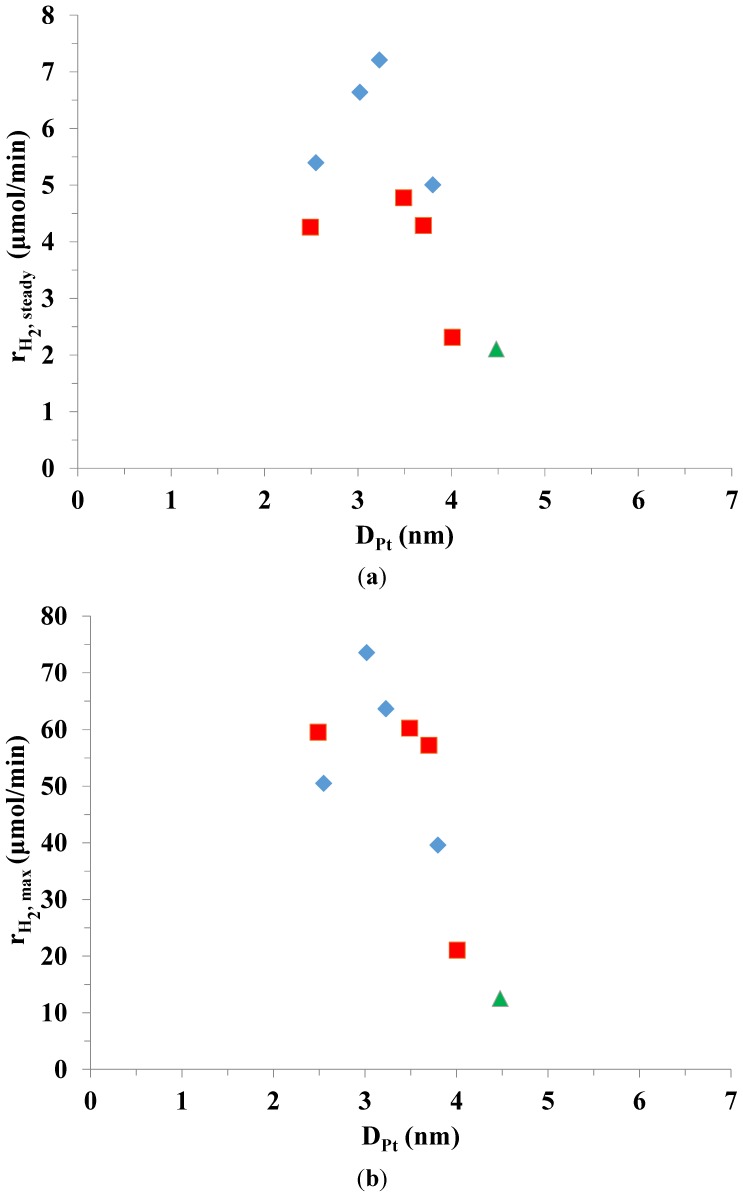
Comparison of H_2_ evolution rates (**a**) steady state and (**b**) maximal as a function of D_Pt_ on photoreduced (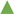) and chemically reduced (CRIS (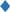) and CRSIM (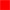)) Pt-TiO_2_ photocatalysts using 50 mM oxalic acid as sacrificial reagent.

With each chemical reduction method, the highest steady state H_2_ evolution rates were achieved with the following catalysts: (i) the 1.25 × 10^−4^ M citrate stabilized Pt-TiO_2_ CRIS sample (*D*_Pt_ = 3.2 nm; *r*_H2,steady_ = 7.21 μmol/min), (ii) the 1.88 × 10^−4^ M citrate stabilized Pt-TiO_2_ CRSIM sample (*D*_Pt_ = 3.5 nm; r_H2,steady_ = 4.78 μmol/min). The correlation between the platinum particle size and the steady state or the initial maximal H_2_ evolution rates are demonstrated in Figure 6. The distribution of Pt particles on the TiO_2_ surface appeared to be more uniform in the samples synthesized by *in situ* chemical reduction method. As a result, CRIS samples had higher hydrogen producing capabilities than the sol-impregnated ones.

At *D*_Pt_ = 3.2 nm average particle size, there was a peak in photocatalytic activity. Smaller Pt nanoparticles are unfavorable, probably due to the loss in metallic character [40,41]. Using photoreduced Pt-TiO_2_ resulted in the lowest photocatalytic activity with the largest Pt nanoparticle size (*D*_Pt_ = 4.5 nm) on the surface. The average Pt particle size on each catalyst and the respective steady state and maximal H_2_ evolution rates are demonstrated in Table 2.

**Table 2:** Average Pt particle size on the prepared catalysts and the respective initial and steady state H_2_ evolution rates and apparent quantum yields using 50 mM oxalic acid as a sacrificial reagent.

Sample	*D*_Pt_ (nm)	*r*_H2, steady_ (μmol/min)	*AQY*	*r*_H2, max_ (μmol/min)
2.50 × 10^−4^ M citrate-Pt-TiO_2_ CRIS	2.6	5.40	1.88%	50.50
1.88 × 10^−4^ M citrate-Pt-TiO_2_ CRIS	3.0	6.64	2.32%	73.54
1.25 × 10^−4^ M citrate-Pt-TiO_2_ CRIS	3.2	7.21	2.52%	63.67
0.63 × 10^−4^ M citrate-Pt-TiO_2_ CRIS	3.8	5.00	1.75%	39.60
2.50 × 10^−4^ M citrate-Pt-TiO_2_ CRSIM	2.5	4.26	1.49%	59.54
1.88 × 10^−4^ M citrate-Pt-TiO_2_ CRSIM	3.5	4.78	1.67%	60.25
1.25 × 10^−4^ M citrate-Pt-TiO_2_ CRSIM	3.7	4.29	1.50%	57.24
0.63 × 10^−4^ M citrate-Pt-TiO_2_ CRSIM	4.0	2.31	0.81%	21.04
Photoreduced Pt-TiO_2_ PROA	4.5	2.11	0.74%	12.50

It was concluded that the most important parameter influencing the H_2_ production efficiency of Pt-TiO_2_ photocatalysts appeared to be the Pt nanoparticle size. As Pt clusters contain different numbers of atoms, the structure and surface bonding is changed which leads to different catalytic activities [42]. The optimal size of the Pt nanoparticles with the best distribution on the TiO_2_ surface was achieved with the CRIS method.

**Figure 7:**
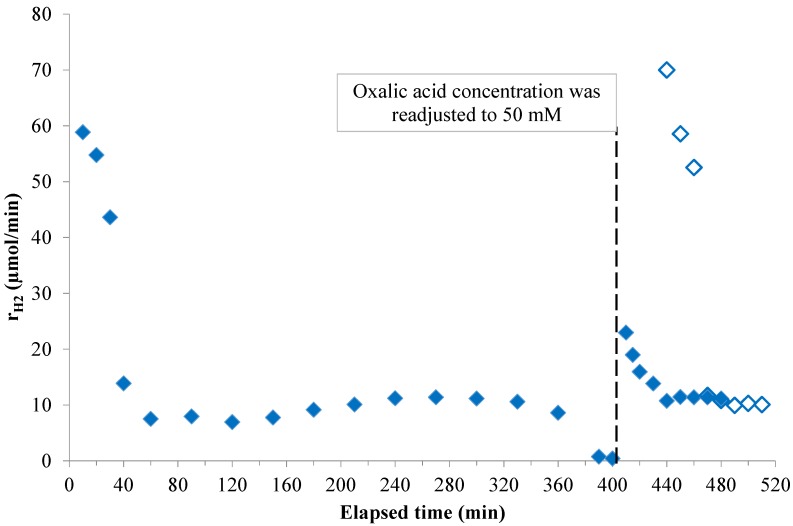
Long term irradiation of Pt-TiO_2_ in the presence of oxalic acid (initial concentration = 50 mM; oxalic acid concentration was re-adjusted after complete mineralization with (◊) and without (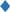) equilibrating the suspension in dark).

To investigate the long term stability of the best performing Pt-TiO_2_ catalyst, the suspension was irradiated for several hours. The H_2_ evolution rate was almost constant until 400 min, when the oxalic acid (*c*_initial_ = 50 mM) totally decomposed. After full mineralization of oxalic acid on the catalyst in about 7 h, the concentration of oxalic acid was re-adjusted to 50 mM in dark. The UV irradiation was restarted after 30 min of stirring and nitrogen purging, in order to provide enough time to achieve the adsorption equilibrium in dark. In the re-initiated reaction the H_2_ evolution rate was almost the same as at the beginning of the experiment with decrease of the reaction rate to the same level after 40 min (Figure 7). In another experiment, oxalic acid was also fully mineralized but the concentration was re-adjusted without switching off the lamps and without waiting for the hydrogen to completely purge out from the suspension and Pt as well. The initial H_2_ evolution rate was about 40% of the original one and it decreased to about the same steady state level that was observed in the first cycle. These results support the plausible hydrogen absorption mechanism and formation of H-electrode described above.

**Figure 8:**
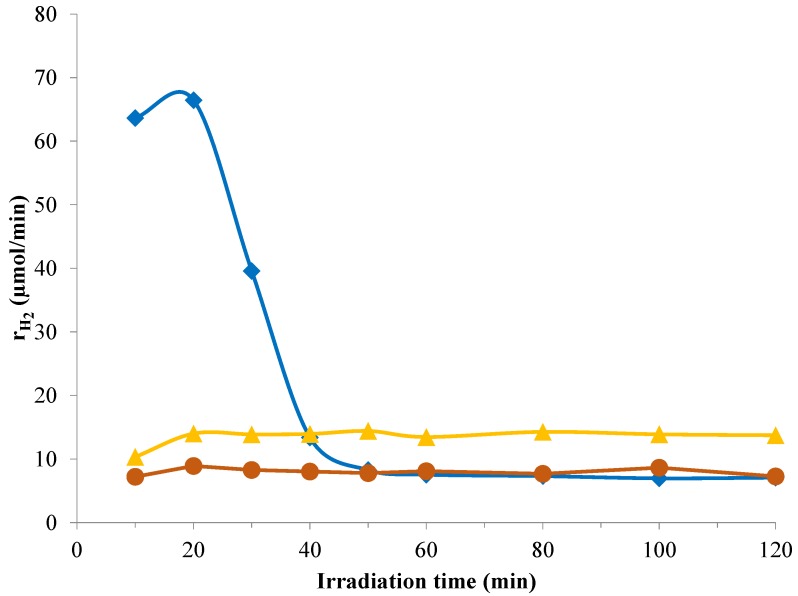
Photocatalytic H_2_ evolution in the presence of oxalic acid using Pt-TiO_2_ (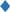) and Au-TiO_2_ with similar average noble metal particle size (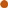) or synthetized by the same chemical reduction method (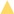).

We also compared the results from our parallel study using Au modified titanias with our best performing Pt-TiO_2_ sample. In one case, the Au nanoparticles were almost the same size as Pt (*D*_Au_ = 3.5 nm, *D*_Pt_ = 3.2 nm), while in another case, exactly the same chemical reduction method was used (*D*_Au_ = 5.7 nm). The optimally sized Au-TiO_2_ catalyst showed an almost constant and (in the steady state) nearly double H_2_ evolution rate than in the case of the above mentioned best Pt-TiO_2_ photocatalyst. It is also noticeable that modifying Aeroxide P25 TiO_2_ with Au or Pt nanoparticles in almost the same size resulted in roughly the same H_2_ evolution rates in 50 mM oxalic acid solution (Figure 8).

In case of Au modified catalysts, initial maximum in hydrogen formation was not observed, indicating no H^+^/H_2_ electrode formation on metallic Au deposited onto TiO_2_.

#### 3.4.3. Decomposition of Oxalic Acid under Anaerobic Conditions

**Figure 9:**
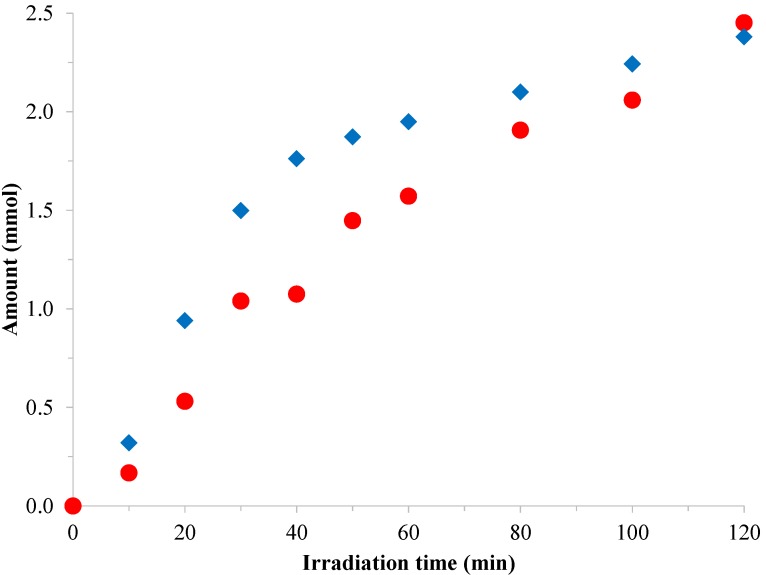
Photocatalytic decomposition of oxalic acid in the solution phase (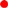) and H_2_ production (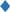) under UV irradiation and anaerobic conditions with Pt-P25 CRIS (*D*_Pt_ = 3.2 nm) photocatalyst.

We investigated the correlation between the H_2_ production and the decomposition of oxalic acid under the same conditions. The residual oxalic acid concentration was determined by HPLC (Figure 9). For this experiment, we used the Pt-modified photocatalyst that performed the best in the H_2_ production measurements (*D*_Pt_ = 3.2 nm, CRIS sample). Correspondingly to the H_2_ evolution, the curve for oxalic acid decomposition is in accordance with hydrogen accumulation. The assumed sum reaction is:

(COOH)_2_ → 2 CO_2_ + H_2_ (1)

After turning on the lamps, the decomposition and adsorption reaches an equilibrium state, so the H_2_ evolution becomes nearly constant. It is important to emphasize that the oxalic acid concentration was measured in the solution phase by HPLC, thus the decomposition of the adsorbed oxalic acid cannot be seen in this representation. It is plausible to think that at the beginning the total decomposition rate of oxalic acid chemisorbed on the surface is significantly higher than the decomposition rate in the solution phase. Therefore, the measured hydrogen evolution rate can be accordingly higher than the oxalic acid decomposition rate in the solution phase. At later stages, the decomposition rate of oxalic acid is nearly the same as the hydrogen evolution rate.

## 4. Conclusions

Differently sized Pt nanoparticles were synthetized onto Aeroxide P25 TiO_2_ by chemical reduction and by photoreduction with constant platinum content (1 wt%). We were able to finely control the size of Pt nanoparticles through the use of different concentrations of the stabilizing agent. Two developed chemical reduction methods (CRIS and CRSIM) were utilized and the efficiency of the catalysts for H_2_ production and the size distribution of surface Pt particles were also investigated. In UV-irradiated O_2_-free suspensions, the Pt-modified TiO_2_ catalysts showed high H_2_ production activities in the presence of oxalic acid. The photocatalytic activity proved to depend strongly on the average Pt particle diameter: there was an optimum in photocatalytic activity at ~3.2 nm average Pt nanoparticle size on the TiO_2_ surface.

In the majority of publications, platinum modified TiO_2_ photocatalysts are used for H_2_ production. However, comparing our best performing Pt-TiO_2_ catalyst with our previous experiments with Au-TiO_2_ prepared by similar chemical reduction method, the performance of Au-TiO_2_ catalysts (with same Au size or made by exactly the same procedure as Pt-TiO_2_) was as good as or even better than the Pt-TiO_2_ when oxalic acid was used as model compound.

The long-term usability of our best performing catalyst was also investigated: until the irradiated suspension contains any easily oxidizable organic compound (e.g., oxalic acid), H_2_ evolution rate remains nearly constant. Considering this result, these catalysts might be utilized effectively for H_2_ production without the loss of photocatalytic activity.

We also investigated the phenomenon of a significant decrease in H_2_ evolution rate at the first stage of experiments when oxalic acid was used as a model compound. It turned out that the formation of H-electrode on platinum might be the key reason of the limited H_2_ generation.
